# The Power of Pets: How Companionship and Social Connections Shape Aging Well

**DOI:** 10.1093/geroni/igaf122.3082

**Published:** 2025-12-31

**Authors:** Marielena Barbieri

**Affiliations:** Mather Institute, United States

## Abstract

As people age, maintaining strong social connections becomes essential for well-being. While pets are often considered loving companions, their impact on older adults’ social and emotional health is not fully understood. This study explores how pet ownership influences loneliness, happiness, and life satisfaction, mediated by social cohesion. Utilizing 4-years of data from the Age Well study (N = 2,863, Mage = 82.31), we examined both the direct and indirect effects of pet ownership on well-being over time. Findings revealed that while pet ownership in 2019 alone had limited direct effects, it significantly enhanced social cohesion during 2020-2021, which in turn reduced loneliness (b = -.29, p < .001) and increased happiness (b = .51, p < .001) and life satisfaction (b = .44, p < .001) by 2022. Differences emerged between pet types: dog owners experienced greater social cohesion (b = .12, p = .001), leading to improved well-being outcomes, while cat ownership showed no significant indirect effects. These results highlight the power of pets—particularly dogs—in fostering social connections that enhance well-being in later life (e.g.,Hajek & Konig, 2020). As loneliness and isolation remain pressing public health concerns among older adults, promoting pet companionship may serve as a meaningful intervention (Holt-Lunstad, 2017; Krause-Parello et al., 2019). This study highlights the need for aging policies and community programs that recognize and support the social benefits of pet ownership, ensuring that more older adults have access to the companionship and connection that can help them thrive.

